# Supplementary material for: Associations of Total Body Fat Mass and Skeletal Muscle Index with All-Cause and Cancer-Specific Mortality in Cancer Survivors
Source: Cancers (Basel). 2023 Feb 8;15(4):1081. doi: 10.3390/cancers15041081 (PMC9953880; doi:10.3390/cancers15041081)
Supplement: Supplementary file 1 [file cancers-15-01081-s001.zip › cancers-2151246-supplementary.pdf]

Supplementary Materials

Associations of total body fat mass and skeletal muscle index with all-cause and cancer-specific mortality in cancer survivors

Table S1. Association between WBFM and risk of all-cause mortality in subgroups of cancer survivors.

Variables	Cancer survivors (N= 1682)		p-interaction
	**No. Death/Total**	**aHR (95 CI) Model 1**	
**All-cause mortality**			
**Sex**			
**Female**	387/737	1.53 (0.92-2.55)	0.001
**Male**	281/945	2.16 (1.15-4.06)	
**Age (years)**			
<65	132/886	2.07 (0.74-5.79)	0.001
≥65	536/796	1.84 (1.20-2.82)	
**Energy intake (kcal/day)**			
<1785	361/668	1.85 (1.04-3.92)	
≥ 1785	256/638	2.28 (1.23-3.85)	0.067
**Comobordity**			
0	384/969	2.21 (1.34-3.64)	0.554
1-4	284/713	1.70 (0.83-3.47)	
**Sex**			
Female	125/737	1.87 (1.11-3.17)	0.303
Male	88/945	1.13 (0.51-2.53)	
**Age (years)**			
<65	58/886	2.01 (0.51-7.97)	0.820
≥65	155/796	1.70 (1.08-2.67)	
**Energy intake (kcal/day)**			
<1785	108/668	1.75 (0.97-3.16)	0.937
≥ 1785	93/638	1.69 (0.91-3.16)	
**Comorbidity**			
**0**	138/969	1.74 (1.03-2.93)	0.961
**1-4**	75/713	1.70 (0.80-3.60)	

Abbreviations: aHR: adjusted hazard ratio, CI: confidence interval.

Model 1: The model adjusted for age, sex, race, education, marital status, smoking status, energy intake, burden of comorbidities, history of more than 1 cancer (for survivors). For cancer survivors, the model was stratified by time elapse since cancer diagnosis. In each set of subgroups, factors used for stratification were not included in the multivariable model.

Table S2. Association between DXA measures of body composition and risk of non-cancer mortality in cancer survivors.

Measures of body composition	No. death/person-years	Age-adjusted HR (95% CI) Model 1	aHR (95% CI) Model 2	aHR (95% CI) Model 3
All-cause mortality				
**Total fat mass**				
Low	111/5318.3	Ref	Ref	Ref
Medium	165/5068.0	1.08 (0.84-1.39)	1.12 (0.88-1.45)	1.13 (0.88-1.45)
High	179/5043.4	1.43 (1.12-1.83)	1.32 (1.02-1.71)	1.33 (1.03-1.72)
**Appendicular skeletal muscle mass**				
Without sarcopenia	170/8627.9	Ref	Ref	Ref
With sarcopenia	99/2422.9	1.44 (1.11-1.87)	1.44 (1.10-1.88)	1.46 (1.11-1.91)
